# Dental Neurology

**Published:** 1901-02-15

**Authors:** W. H. Whitslar

**Affiliations:** Cleveland, Ohio


DENTAL REGISTER.

Vol. LV. February 15, 1901. No. 2.

### DENTAL NEUROLOGY.

BY W. H. WHITSLAR, M.D., D.D.S., CLEVELAND, OHIO.

Read before the Ohio State Dental Society, December, 1900.

The composition of man leads one to think that there will ever continue a ceaseless study of mankind. An army of investigators has given to the world a fund of knowledge which unfolds to those of the present time data upon which every effort is being made to widen perceptably the great truths of nature and make life simpler. Toward these conclusions, however, we find that the means of elaboration have been far from simple. Man has been dissected, every part chemically analyzed, physiologically studied, surgically removed from all but the tender threads of life, and psychical relations have entered into the common fund of the existence of our knowledge of him. It is apparent, too, that the morbid conditions are factors in the establishment of facts which are monitors to the human race.

We anatomize man to study the structure and economy of his body, analyze by chemical processes the relations he sustains to the earth and how he uses the product of it for his own sustenance, epitomize these two subjects and call it physiology, thus leading to the highest senses that a human body is capable of realizing, namely, those of sight, hearing, touch, taste, pressure and temperature. Some of these senses may become impaired, and there is still remaining an incomparable something which is called "mind."

The reasoning and intellectual faculties as a whole constitute mind. From very early times the brain has been believed to be the seat of all that we mean by consciousness. It is only within recent times that cerebral physiology has demonstrated that a particular sensation is connected with a certain anatomical area. Every thinking human being is concerned in the cerebral processes that lead to sensations, movements, emotions and intellectual acts. How do these commence and finally cease? It is believed that molecular changes are translated into consciousness and intellectual acts, but how? It is a matter of speculation. We can assert, however, that there is a continuous force transmitted from parent to offspring, bringing with it the inherent property which takes cognizance of co-existing things. The theories of heredity would place this conveyance within the material known as somatoplasm, idioplasm or chromatin, as designated by different theorists. Further than this anatomy or chemistry can not fathom the secret of transmission of matter. The whole proposition hinges upon the true significance of the word, "Life." Now, the means by which an animal knows that which is going on about it, the carrying of information to some central body, and putting itself into relationship with the surrounding medium, is called the nervous system In consequence of the relationship to the outer world the nervous system is primitively superficial, and is closely related to the outer layers of the body.

In the very lowest orders of animal life we find sensitivity without a nervous system. The amœba is sensitive to stimuli and changes the position of its protoplasm because of the irritation. As we ascend in the scale of the animal kingdom we find the nervous system is more or less distributed throughout the whole body.

Without discussing the variability in the distribution of the nervous system throughout the gradual rise in the scale of life, let us inquire into the composition of it in the human being. The essential features of a nervous system are central cells and conducting fibers, and as the organism develops we perceive that cells and fibers become aggregated so that we have definite centers or lines along which nervous impulses will pass. The most important of these masses form the brain or encephalon, the most important fibers the nerve-cords, then there are secondary masses called ganglia and which are connected with the former. Now, information comes from without by a set of specialized cells, sense cells, belonging to the epithelial regions of the body, and these are derivitives of the epiblast. These are peripheral organs in a certain sense. The widest distributed sense cells are the tactile cells, more complex are those concerned in the sense of taste, and still greater are the higher senses of sight, smell and hearing. With the full development of the brain these organs send to it messages from the outer world, and these are converted into the sensations peculiar to the sense organ from whence they came. These nerves which carry impulses toward the central nervous system are designated as the afferent or centripetal, while the nerves that send impulses from the brain to the peripheral nerves are the efferent or centrifugal terminations of neural action.

It is impossible to tell what a nerve impulse is, whether it is a wave of chemical change, or a molecular change, and so far as we know, the only function of the nerve fiber is to conduct impulses from nerve centers to peripheral organs, and *vice versa*, or from one organ to another. This neuricity or nerve force has actually been measured, and its velocity Helmholtz found in the human arm to be from 30 to 90 metres per second. These were motor impulses, but there is no reason to believe that sensory impulses do not travel at the same rate.

Diseases and other conditions may modify the rate of impulse, as for instance, heat increases conductivity, whereas cold diminishes nerve impulse. The change of condition in a nerve may be modified during the application of a current of electricity. A decrease of the electro-motive force in a nerve results when the current is in the opposite direction to the nerve current, and the same force is increased if it is in the direction of the flow of the nerve current. These conditions are designated the negative and positive phases of electrotonus.

Paralyzant action of nerves may be produced by inhibiting the electro-motive force that is opposing the natural nerve current, or reducing the natural power to a state where it is easily overcome by the unnatural forces. This nerve weakness which is caused by exhaustion of nerve centers is called neurasthenia.

Like all tissues of the body, excepting in parts of the teeth, the nerves require nutrition to maintain health and proper existence. The nutrition of nerves is dependent upon the nerve cells, which are a part of the fiber in its course, or rather, the fiber is morphologically a process of the cell. There is a nutritive unity between the fiber and the cell. If a nerve is divided and not kept artificially separated regeneration occurs. This consists in the outgrowth of new axis-cylinders in the form of fine fibers from the ends of the divided axis cylinders of the central stump of the nerve. These push their way into and along the degenerated fibers and develop into complete nerve fibers

Surgery has taught us that peripheral nerves may be brought together, sutured, and their function restored. As early as 1836 Baudens performed nerve suture, but with negative results. In 1863 Nelaton revived the procedure. Modern surgery, owing to antisepsis and better technique, has increased the percentages of successes in the operation. This led an eminent surgeon to remark that "The surgeon who neglects to suture a divided nerve commits the same mistake as he who neglects to reduce a fracture or fails to unite a divided tendon." Many authors have expressed their doubts as to the possibility of regeneration of brain tissue, and others have gone to the other extreme and claim that complete repairs can take place. An eminent physiologist states that "It is a remarkable and as yet unexplained fact that regeneration of the fibers of the central nervous system, at least in the higher animals, does not occur."

It is very apparent that regeneration of tissues is due to the nutrition of the parts, and the proper nutrition of nerves is dependent on their connection with nerve cells. It has been stated that all tissues are provided with "trophic nerves," which regulate the nutrition of the organs which they supply. Evidence disproves this doctrine. Quoting from Professor George N. Stewart, an eminent authority on physiology, he says: "It is true that division of the trigeminus nerve within the skull is sometimes followed by cloudiness of the cornea going on to ulceration, and ultimately inflammation and destruction of the eyeball. Ulcers form on the lips and on the mucous membrane of the mouth and gums, and the nasal mucous membrane of the same side correspondingly as the divided nerve becomes inflamed. But in this case the sensibility of the eye is lost, and reflex closure of the eyelids ceases to prevent the entrance of foreign bodies. The animal is no longer aware of the contact of particles of dust or bits of straw or accumulated secretion with the conjunctiva, and makes no effort to remove them. The lips being also without sensation, are hurt by the teeth, particularly as the muscles of the mastication on the side of the divided nerve are paralyzed, and decomposed food, collecting in the mouth, and inhaled dust in the nose, will tend still further to irritate the mucous membranes. There is thus no more need to assume the loss of unknown trophic influences in order to explain the occurrence of the ulcerative changes than there is to explain the production of ordinary bed sores, bunions or corns on parts peculiarly liable to pressure. And, as a matter of fact, if the eye be artificially protected after section of the trigeminal nerve, the ophthalmia either does not occur or is much delayed."

Also Professor Stewart says: "The nutritive alterations in muscles and salivary glands after section of motor and secretory nerves seem to depend on functional and vaso motor changes. In muscles they come on far too late to be due to the loss of 'trophic' nerve fibers."

Now, these statements lead us to conclude that centrifugal nerves are, in their functional activities, especially of the vascular system, dominated by the vaso-motor system of nerves. This system of nerves, divided according to its uses, indicate that the vaso-constrictor nerves preside over the motor fibers and the vaso-dilator over the inhibitory influences. In all of these, also including the trophic nerves, there is a unity of action, and where there is a deficiency in one part it affects the normal activities. It is for these reasons, then, that the study of the physiology of the nervous system should be understood ere we are able to comprehend the pathology of it.

The fifth cranial nerve, called sometimes the trifacial, or trigeminous, resembles a spinal nerve, being compound in its function and arising from two roots with a ganglion upon its posterior root. This nerve and all of its dependencies are therefore subject to its excitement or depression by vaso-motor or trophic changes as well as the so-called sympathetic relations with other nerves. It is easy to perceive thus that other organs than those contained in the mouth are subject to the derangement due to dental origin. This would include most frequently the eyes and the ears. Affections of the larynx, alimentary canal, heart, and even uterus, arise because of dental irritation. Authentic cases of hip disease are on record related to dental disease. Indeed, various cerebral disorders may ensue, such as insanity, epilepsy, hysteria and headaches, all of which may be proven by authentic cases.

The most common result of dental irritation or neurosis is local odontalgia, due to diseases of the dental pulp, which may be superinduced even by systemic influences. I have known anemia to result in a persistent dental neuralgia, to be relieved only by enriching the nutrition of the blood. Professor Flagg reported a case of neuralgia due to malaria poisoning.

Syphilis may have its share in producing pain when the teeth are associated with periostitis of the jaws, and no doubt the nerves of the dental pulp are affected by the circulation of intoxicated blood. Active contractions of the facial muscles are produced by nerve reflexes, and are known as spasms of the muscles, trismus and torticollis. Paralysis and tetanus have left records of a previous history of carious teeth. Examples of these are frequently found in literature. A year ago it was my pleasure to restore the free use of an arm by the discovery and removal of the cause; i. e., a putrescent dental pulp in a lower third molar, to which one end of a gold bridge was attached.

One of the most common results of diseases of the teeth, aside from odontalgia, is facial neuralgia or prosopalgia. The term neuralgia (nu-ral-je-ah) literally means "pain in a nerve." It is a term used to designate something we do not understand, excepting as a disagreeable sensation. Pain is only one of the phenomena of reflex irritation.

In all of these conditions above related, age, sex, heredity and climate are important predisposing causes of neuralgia.

The seriousness of dental neuralgia is made clear to us when it is remembered that cerebral difficulties follow dental disturbances. Headache is a common example, but epilepsy, hysteria and insanity are the distressing ones. There is a class of humanity that is deficient in nerve function, particularly of the central nervous system, induced by traumatic or hereditary influences. These individuals are apt to succumb easily to mental or other nervous diseases. Primarily, however, these diseases might occur without external influences, having in the course of development of the body been pathologically predisposed to this class of diseases. In other cases, eternal influences, such as mental work, sorrow, care, psychical irritation and disease give rise to an outburst of pathologic brain or spinal function.

It is a matter of common experience among dentists to notice individuals suffering intensely with dental irritation, that the borderland of insanity has almost been reached. It must be conceded that in most of these instances there is a condition or phase which might be called a neuralgic diathesis, in which depression, or lack of nutrition, superinduces lack of co-ordination among the sensory cells of intelligent design. Thus perverted nutrition is influenced by nervous excess, emotionality, intellectual strain and diseases of the teeth occur as well as lack of their proper development.

In conclusion, gentlemen, while dental neurology comprehends the anatomy, physiology and diseases of the dental nerves, it is my conception that it should include those psychical relations which are so often observed between dentist and patient. The length of this paper precludes, however, the discussion of this phase of the subject at this time.

DISCUSSION OF DR. WHITSLAR'S PAPER.

Dr. H. A. Smith: From former papers read by Dr. Whitslar I have been impressed with the fact that he is a most excellent physiologist, and the paper to-day convinces me that he is also well up in pathology.

A lady once said to me, "What is the reason that dentists are not able to control pain? Why don't they treat these symptoms more?" It was a poser to me. Are we not taught to relieve pain? But the question is not are we not taught to do this, but do we relieve pain? If we do not it is because we are not good physiologists; we do not understand our physiology, and certainly we can not be pathologists and treat nerve pain unless we do know physiology and pathology.

It is not very good form to cite cases, but we all of us have such cases. This case was one of odontalgia, presented by a man who was not certain as to location of it, but thought it was in the lower jaw. On taking his seat in the chair he said, "The pain has gone to my large toe." Thus it went from one place to the other. The irritation was primarily in the large toe, and through reflex neurosis affected the teeth, as there was no apparent cause present in the teeth.

There are many cases where pain is reflected to the teeth from the eye and ear. For a long time oculists did not believe that lesions of the teeth would affect the eye, but more lately they have come to recognize the truth. In 1885 there was quite a prolonged discussion between a Dr. Sexton, of New York, and Dr. Harlan as to the advisability of saving devitalized teeth. Dr. Sexton had fifteen hundred cases where diseases of the ear were caused through devitalized teeth. Dr. Harlan claimed it was not necessarily the cause. But there is no doubt that a tooth without a pulp is at times a source of irritation, and Dr. Sexton claimed this was so, although not always recognized by the individual, and Dr. Harlan to the contrary.

The question now comes up as to what conditions of the pulp may cause it? Is it a hyperemic or suppurative condition of the pulp, or is it caused by peridental disease of some kind? It is probably a pulp in which there is a hyperemia which induces reflex action in the organ of hearing. When there is caries we may have more or less irritation of the pulp, and this could cause earache reflexly. So it seems that Dr. Harlan was correct in his statement.

Headache is also very frequently the reflex result of diseased teeth. I wonder if we realize what a great deal of discomfort of life arises from headache? If we did there would be much more care on our part.

An English gentleman—a Dr. Hilton, I think—in diagnosing headaches produced reflexly from the teeth, claims, if it is an occipital headache, the anterior teeth of maxilla are affected; if the headache is temporal, it is invariably the upper teeth. These are well-established facts. I recall a case where the reflex from diseased teeth affected the sight.

A teacher had excessive lachyrmation; she was kept from her occupation by the excessive flow of tears. It was more profound in the left eye; she could scarcely see. On that side of the mouth we found a tooth the pulp of which was exposed, but still alive. On the proper treatment of this tooth the flow of tears ceased in three weeks. The other eye was not so bad, only slight lachrymation ; but there was also a lateral incisor which was abscessed ; on treating this the flow in that eye ceased in about the same time. I can not discuss this paper, but have simply stated a few cases. I will be very glad if the Doctor, in a concluding paper, would go into the discussion of the psychological relation of ourselves to patients. I think we are indebted to the essayist for so able a paper, and I only regret I could not give it more attention.

Dr. Otto Arnold : The essayist has presented to us an epitome in neurology which contains valuable food for thought. He has shown us something of the magnitude and complexity of the nervous system ; also its susceptibility to extensive disturbances from slight causes. He has confined himself to a statement of theories without taking a positive stand in any of the points at issue, and left the practical application of the subject to others. In the application of this subject to the practice of dentistry I need only remind you, without entering into details, of the familiar clinical facts—the exhibition of pain and discomfort beyond the point of actual injury. Indeed, it is a very well accepted mode of procedure in cases of neuralgia about the face and cranium to search cautiously for exciting causes within or about the teeth, such as exposed pulps, putrid canal, pulp stones. There may be irritation from large fillings, etc. Neural disturbances from dental irritation may and do manifest themselves in a variety of ailments among children during the eruptive period, due to irritation at apical ends of the developing teeth. This cause, I fear, is not always recognized by the general practitioner. Again, the result of dental irritation may be a neurasthenia, leading to insanity. These latter and more serious conditions are seldom seen by dentists, except in the cases referred to them by physicians who recognize such relations, or under other unusual circumstances, as in the following instance.

Some years ago my services were sought to relieve a number of insane patients who were suffering from well-defined dental affections. After operating for all that were known to require such services, the attending physician directed my attention to a good-natured looking woman of about thirty years of age, who had been present throughout my operations, and seemed an interested but speechless spectator, and had been an inmate of the hospital about two years. The doctor, more in jest than otherwise, asked her if she did not also wish some teeth extractsd. To our surprise, she nodded assent. I proceeded to examine her mouth, and was again surprised at what I saw. The upper molars on both sides were loose with abscesses of long standing, that had invaded the tissues in all directions. Extraction was resorted to, which was followed by the discharge of large quantities of offensive pus, and altogether this was the worst case of the day. The remarkable fact in this case was that she had never complained nor shown any unusual symptoms associated with such disturbances. I did not again see this case, but was informed at intervals of her improvement and final dismissal as cured of her mental ailments in less than a year. May we not at least conjecture that the dental trouble was an important factor in this case?

Dr. Wright: I have been very much interested in Dr. Whitslar's paper. I have heard several of his papers, and they have always been of this high class. During the time of its reading there were several things I thought I would like to know more about. Psychology for one. Another question in regard to tropic nerves —whether simply vaso-motor or trophic in their influence? Another, is this question of neuron.

In surgical operations, where there has been a division of a large nerve, can it be regenerated? It seems to me we are still in the dark on this subject. Of course, the connective tissue surrounding these nerves is easily and promptly repaired, but whether or not the living nerve is regenerated is still a matter ot some doubt. We have seventy-six millions of people in the United States. Some neurologists have said that in the human brain of an adult there are some seven hundred millions of nerve cells. We can readily see what a terrible thing we have to study if we are to know all about them. Fortunately for us, they are arranged in bundles or columns, a great many acting toward the same end, and we can thus more easily get at their actions.

Dr. Smith referred to these reflex troubles. Richardson also made valuable studies in this direction. One case of mine: I extracted a bicuspid root for a man, which did not hurt as far as I was concerned at all; but he curled himself up and complained of pain in his ear. This was rather strange, and as he was an ear specialist I asked him to make some study of it and give me the results, but nothing definite was ever ascertained.

Dr. C. R. Butler: It occurs to me that, notwithstanding this subject is set down as being one of the most important, that we dentists should understand and know all about, at the same time we must be careful not to theorize too much. All who attend the societies regularly know that there are men who are always presenting some wonderful ideas in papers, but which are practically useless. We want something more practical; we must have a foundation to stand upon. But the two papers just read, which are much alike, are not of this order; they are fundamental, and in order to be intelligent practitioners we must know the fundamental principles in the chemical, physiological and pathological phenomena. You often hear such question as this, "This physiological chemistry—what in the world does it amount to, and what is the necessity of it?" I wish I knew more about it.

We occasionally have papers of this kind, and, notwithstanding they are beyond the reach of most of us, yet they are most valuable, for they set us to thinking and studying. One point I would like to ask Dr. Whitslar: How can we have irritation if the subject is not sensitive? There was a sort of play of words along there on the second page of this paper, I thought. That we can have irritation where there is no sensation is something of which I was not aware.

Dr. George H. Wilson: While the Doctor was reading his paper the thought came to my mind, Here is an illustration of a point brought out in our President's address, the thought of impressing upon students entering into the study of dentistry of its high professional nature. Should we not, when we give such instruction, impress upon their minds that the work they are going into is that of a learned profession, and enforce upon them the necessity of a thorough knowledge of chemistry, bacteriology, physiology, etc., instead of letting them believe that it is purely mechanical, and when they have a thorough mastery of this it is all that is necessary? They should possess as thorough an education as the medical practitioner. This will therefore necessitate our colleges and general profession enforcing these studies, and when it comes to the four-year course I think it will be done.

Dr. H. A. Smith: Dr. Wilson has suggested a thought which is well worth noting. I wish to say just a few words to correct the thought in the minds of the laity that dental colleges are negligent in the teaching of these branches. In the college with which I am connected we have a society, formed of the junior and senior classes, for the purpose of discussing various subjects pertaining to the practice and science of dentistry. This very subject under discussion is an important one before them: "Is the study of chemistry important for the dental profession?" They form into two sides and debate the question. When they take up such a subject for discussion I think it shows that they realize the necessity for a knowledge of these subjects.

Dr. Wilson: I did not mean to say that the schools do not teach such subjects, but that the general profession should advocate the necessity for it, so that when students go to college they will not think them superfluous. We should educate them to believe that they are fundamental, and that their future success depends upon them.

Dr. W. H. Whitslar: About one hundred years ago Galvani, the great scientist, studying the electrical frogs, suspended some by a copper wire from an iron rod on his balcony, and he observed that when the wind blew the frogs against the iron rod there was a peculiar twitching, and he concluded that there was some kind of an electrical current developed within the frogs.

At the same time Volta, experimenting with metals, found, by taking two metals of different kinds, zinc and copper, for example, and bringing them into contact in a fluid they created an electrical current also. He repeated Galvani's experiment, and concluded that Galvani was mistaken in his ideas, and that there was no electrical action in the frog, but the action was produced by bringing into contact the copper and iron metals. The flesh of the frog acted just the same as the fluid between the two metals. Consequently he concluded that there was an electro-motive force in the muscle nerve of the frog. The question then arises, Is it a molecular or chemical change? This is not decided as yet. There must, however, be some chemical change. Take, for instance, the nerves of the spinal cord; they are about 60 percent water, and the remaining percent is proteid, etc. And it stands to reason that there must be some chemical reaction between these elements which causes these neuralgias.

We do know that there is some kind of a current passing back and forth along the nerve, stimulating the muscle, and it would also seem possible that there should be a kind of electrical current passing from one person to another. Example: At the Tri-State meeting at Put-in-Bay there was an old practitioner present, and, with a number of other young men, I gathered around him to listen to some of his doctrines. He was asked how he controlled his patients. He grasped a young man by the hand and looking him in the eye, said, "This is the way I get hold of the patient first, and I am then able to control him in the chair." It may be hypnotic power; it is at least psychological. We have such an example here to-day. The doctor here says I am a good physiologist, yet I know I am not. I have tried to study this simply that I might be able to better control my patients. Students to-day do not give the proper attention, they do not realize the good to be derived from the study of physiology. It is one of the paramount principles in the science of dentistry. We are constantly studying physiology in our offices, and if we were not we would not be able to become proficient practitioners.

